# iRGD Tumor Penetrating Peptide-Modified NK Cells Exhibit Enhanced Tumor Immune Infiltration Ability and Anti-Tumor Efficacy

**DOI:** 10.2174/0109298665348639250115113650

**Published:** 2025-02-07

**Authors:** Ge Song, Xueyong Qi, Yi Zhao

**Affiliations:** 1 School of Pharmacy, Jiangsu University, Zhenjiang 212013, Jiangsu, China;; 2 Jiangsu Topcel-KH Pharmaceutical Co., Ltd., Nanjing 210093, Jiangsu, China;; 3 Department of Medical Oncology, Nanjing Tianyinshan Hospital Affiliated to China Pharmaceutical University, Nanjing 211199, Jiangsu, China

**Keywords:** NK cells, solid tumor cells, iRGD, immunotherapy, MCTSs, TPP

## Abstract

**Background:**

Natural killer (NK) cells, as part of the group I innate lymphocytes (ILCs) are essential for tumor immune surveillance. NK cells can recognize and eliminate target cells without the need for prior sensitization or restriction of major histocompatibility complexes (MHCs) and antigens. However, the limited infiltration of metastatic NK cells poses significant challenges for advancing adoptive cell immunotherapy for solid tumors.

**Objective:**

This study aimed to explore the potential of using tumor penetrating peptide (TPP) iRGD to promote the delivery of activated NK cells to deeper layers of tumor tissue.

**Methods:**

Flow cytometry was performed to evaluate the activation, inhibition, and expression of other receptors involved in cytotoxicity. High-pressure liquid chromatography (HPLC) and mass spectrometry were used to detect the purity of iRGD. 1,2-distearoyl-sn-glycero-3-phosphoethanolamine-poly(ethylene glycol)-iRGD (DSPE-PEG-iRGD) was synthesized. Surface modification of cells was performed using DSPE-PEG-iRGD. Multicellular tumor spheroids (MCTSs) were established to evaluate permeability. In addition, in order to better simulate the physiological characteristics of solid tumors *in vivo*, we generated 3D spheroids from HGC27 gastric cancer cell line and BXPC-3 pancreatic cancer cell line to study the anti-tumor effect of NK cells with combination iRGD *in vitro*. The mouse models of gastric cancer and pancreatic cancer were used. In addition, the synergistic anti-tumor effects were evaluated *in vivo* based on the tumor volume and body weight of mice.

**Results:**

Initially, we treated NK cells with interleukin-2 (IL-2), resulting in significant activation as indicated by upregulation of CD56. On the 15th day, the proliferation of CD3-/56+cell population in NK cell culture containing IL-2 significantly increased, and the NK cell amplification factor was greater than 300. In addition, NK cells exhibited increased cytotoxicity towards cancer cell lines. When the ratio of effect to target was 10:1, the killing rate of NK cells against BXPC-3 was 83.1%. iRGD modification enabled NK cells to penetrate MCTSs, resulting in cytotoxicity against target HGC27 and BXPC-3 cells. In addition, NK cells modified with iRGD significantly reduced tumor growth in the xenotransplantation model of gastric cancer and pancreatic cancer mice model.

**Conclusion:**

In summary, our results indicated that NK cells exhibited higher efficacy and lifespan against cancer cell lines *in vitro*. Furthermore, the integration of iRGD into NK cells led to improved infiltration and targeted elimination of MCTSs. Moreover, the application of iRGD-modified NK cells has shown significant anti-tumor efficacy against solid tumors *in vivo*. This joint strategy may significantly improve the efficacy of NK cell immunotherapy in treating various solid tumors.

## 1 INTRODUCTION

Cancer is a major disease that affects human health and is the leading cause of death worldwide. According to the World Health Organization (WHO), nearly 10 million people died from cancer in 2023 [1]. Among the top 10 cancers, the incidence rate of 6 cancers continues to rise, including breast cancer, prostate cancer, uterine body cancer, pancreatic cancer, oropharyngeal cancer, liver (female) cancer, kidney cancer, and melanoma, as well as colorectal cancer and cancer in young adults [2]. By 2030, it is estimated that the global population will approach 8 billion, and the burden of cancer on the world will become increasingly heavy. Cancer is a major public health challenge faced by China. In 2020, the country reported 4,546,400 new cases of cancer and 2,992,600 cancer-related deaths, accounting for 25.1% and 30.2% of global cases, respectively [3]. With the relaxation of policies by relevant regulatory agencies and the continuous advancement of biomedical technology, immunotherapy has become a key element in cancer treatment.

Adoptive cell therapy (ACT) is one of the most important types of immunotherapy [4]. It involves infusing immune cells, such as tumor-infiltrating lymphocytes or peripheral blood-derived T lymphocytes (T cells) and natural killer cells (NK cells), into the patient’s body [5]. NK cells are a part of group I innate lymphocytes (ILCs) and play a crucial role in tumor immune surveillance [6]. Unlike specific T cells, NK cells can recognize and destroy target cells without prior sensitization or dependence on major histocompatibility complex (MHC) and antigen presentation [7]. Due to this unique ability, NK cell-based immunotherapy has gained momentum in cancer treatment. For example, the expression of interleukin-21 (IL-21) significantly enhanced the cytotoxicity of NKG2D CAR-NK cells towards lung cancer cells in a dose-dependent manner, and effectively inhibited tumor growth *in vitro* and *in vivo* [8]. In addition, depletion of NK cells promoted liver metastasis of lung cancer cells [9]. However, the clinical efficacy of NK cells was limited by their poor infiltration into solid tumors [10].

The iRGD peptide (sequence: CRGDKGPDC) can enhance the permeability of tumor blood vessels, and promote internalization and penetration into tumor tissues, thereby improving diagnostic accuracy and therapeutic efficacy [11-13]. The mechanism behind this process is believed to depend on the RGD domain and CendR motif iRGD [14]. It follows a multi-step and tumor-specific mechanism. Firstly, iRGD binds to the elevated integrins αvβ3/β5 on tumor vascular endothelium and various tumor cells, and is then hydrolyzed and cleaved by cell-surface-associated protease proteins to produce CRGDK/R, exposing the CendR motif. The truncated peptide loses its affinity for the primary receptor integrin and binds to neuropilin-1, triggering the tissue infiltration pathway [15]. Shao *et al.* reported that iRGD could improve the infiltration of metastatic T cells into tumors [16]. Specifically, iRGD-coated T cells showed a significantly enhanced ability to penetrate deep into tumor tissue.

Therefore, this study aimed to investigate whether iRGD-modified NK cells can promote immune infiltration in solid tumors and perform functional verification at the cellular experimental levels. This may provide new ideas for tumor immunotherapy research and new technical strategies for clinical trials.

## 2 MATERIALS AND METHODS

### 2.1 Cell Culture

Human cancer cell lines HGC27 (gastric cancer), MGC-803 (gastric cancer), BXPC-3 (pancreatic cancer), BT-549 (breast cancer), HLA-A24 positive SNU719 (gastric cancer) cells, and NIH3T3 (embryonic fibroblast) cells were purchased from the cell bank of Shanghai Institute of Biochemistry and Cell Biology, and grown in RPMI-1640 medium supplemented with 10% fetal bovine serum (FBS, Gibco, USA), 100 U/mL penicillin, and 100 μg/mL streptomycin at 37°C and 5% CO_2_.

### 2.2 Isolation and Expansion of Primary Human NK Cells

According to the research plan authorized by the Shanghai Blood Management Bureau, human peripheral blood mononuclear cells (PBMCs) were sourced from the Shanghai Blood Center. According to the established protocol, fresh or frozen PBMCs were cultured in RPMI-1640 complete medium (Hyclone, USA) for 2 weeks and stimulated with IL-2 to promote NK cell expansion [17]. The culture medium was replaced with fresh RPMI-1640 and IL-2 every 2 days. Then, NK cells were purified using CD56-positive selection magnetic beads (Miltenyi Biotec, Germany).

### 2.3 Flow Cytometry

On the 0^th^ and 14^th^ day of cultivation, 1.0×10^6^ NK cells were analyzed by flow cytometry. The cells were labeled with appropriate fluorescent conjugated antibodies targeting CD3 (HIT3a, BD Bioscience, USA), CD16 (B73.1, BD Bioscience), CD45 (UCHL1, BD Bioscience), CD56 (B159, BD Biosciences, USA), and CD69 (BD Bioscience, USA) to monitor NK cell subpopulations and activation status. The cells were then stained using standard flow cytometry methods and harvested; they were then analyzed using CellQuest (v2.1) software (Becton Dickinson) employing a FACSort flow cytometer [14]. All experiments were repeated at least three times.

### 2.4 *In vitro* Cytotoxicity Assays

For cytotoxicity assay, 5.0×10^6^ NK cells and BXPC-3 cells (a representative of solid tumor cell line) were co-cultured in a real-time cell analyzer (RTCA S16, Agilent, China) with an efficiency target ratio (E:T) of 10:1. The cell killing ability and killing ratio were estimated based on the data detected by the instrument after about 4 hours to determine cell toxicity [18]. All experiments were repeated at least three times.

### 2.5 Synthesis of 1,2-distearoyl-sn-glycero-3-phospho-ethanolamine-polyethylene glycol-iRGD (DSPE-PEG-iRGD)

The cyclic peptide Ac-CCRGDKGPDC-NH2 (C-iRGD) has an additional cysteine at the C-terminus of iRGD, while the control peptide Ac-CCRGEKGPDC-NH2 (C-iRGE) and iRGD are conjugated with FAM at the N-terminus, which were purchased from Ruixi Biotechnology Co., Ltd. (China). DSPE-PEG-maleimide (DSPE-PEG-Mal) was derived from Laysan Bio, Inc. (USA). Before synthesizing the target structure, the purity of iRGD was first detected using HPLC and mass spectrometry. As mentioned earlier, DSPE-PEG-Mal and C-iRGD were mixed in a 1:1 molar ratio in Hepes buffer (pH=6.5) and gently stirred at room temperature in a nitrogen atmosphere for 48 hours. Afterward, the obtained reaction mixture was placed in a dialysis bag (molecular weight cut-off=3500 Da) and dialyzed in deionized water for 48 hours to remove any unreacted iRGD. The final solution was then freeze-dried in the dialysis bag and analyzed using matrix-assisted laser desorption/ionization-time-of-flight mass spectrometry (MALDI-TOF-MS) and ^1^H NMR spectroscopy [19].

### 2.6 Surface Modification of Cells Using DSPE-PEG-iRGD

1×10^6^ NK cells were resuspended in PBS and incubated with 20 μg of DSPE-PEG-iRGD at 37°C for 30 minutes to prepare iRGD-coated NK cells. Then, after iRGD modification, the morphology of the cells was examined under an optical microscope. The iRGD-coated NK cells were washed with PBS 3 times, and then centrifuged (180×g, 5 min at 37°C) to remove unreacted reagents. Then, flow cytometry was used to analyze the proportion of NK cells modified with DSPE-PEG-iRGD-FAM [19]. All experiments were repeated at least three times.

### 2.7 Penetration and Growth Inhibition on Multicellular Tumor Spheroids (MCTSs)

HGC27 and BXPC-3 cells were inoculated in a 96-well round plate (Corning, USA) with ultra-low attachment surface, and cultured in RPMI-1640 medium supplemented with 10% FBS (500 in 150 μL). Daily observations were performed under an optical microscope to monitor the formation of spherical bodies and measure their diameter using ImageJ software. Once the diameter of the spheres reached about 200 µm, they were selected for further research. To investigate the infiltration of NK cells into MCSs, the NK cells were first labeled with CFSE (carboxyfluorescein succinimidyl ester, Abcam, UK) in PBS at 37°C for 10 minutes, and then modified with DSPE-PEG-iRGD (20 µg modification reagent per 1×10^6^ cells). The CFSF-labeled NK cells, NK cells with DSPE-PEG-MAL, and NK cells (in combination with iRGD or modified with DSPE-PEG iRGD) were added to spheroid culture and incubated at 37°C for 20 hours. Cytotoxicity assays were performed for 48 hours by exposing MCSs to NK cells, NK cells bound to DSPE-PEG-MAL, NK cells bound to iRGD, or NK cells modified with DSPE-PEG-iRGD in the E:T ratio of 10:1. Then, MCSs were incubated in fresh medium for another 24 hours. The diameter and morphological changes of the spheres were evaluated using a bright-field microscope (Leica, Germany) [20]. All experiments were repeated at least three times.

### 2.8 Xenograft Mouse Models

The ethics committee of Jiangsu University approved all experiments in this study. For subcutaneous tumor models, 50 6-8 week old male BALB/c nude mice purchased from Vital River (Beijing, China) were subcutaneously injected with HGC27 and BXPC-3 cells (5 × 10^6^ suspended in 100 μL PBS). Two weeks after tumor induction, mice were intravenously injected with 0.1 mL PBS, NK, DSPE PEG ML NK, iRGD NK, or DSPE PEG iRGD NK every 7 days, for a total of 2 times. During adoptive cell transfer, the tumor development was examined in mice. The tumor volume and tumor weight were recorded (the tumor volume was recorded every 3 days and the tumor weight was measured on the 18^th^ day).

### 2.9 Statistical Analysis

GraphPad Prism 6 (GraphPad Software, San Diego, CA) was used for all statistical analyses. Unless otherwise specified, all values have been expressed as mean ± standard deviation (SD). Student’s t-test (2 groups) and one-way analysis of variance (ANOVA), followed by Tukey’s post-hoc test (multiple groups), were employed to analyze normally distributed data. Shapiro Wilk test and Levene test were conducted, respectively, to test the normality and homogeneity of inter-group variance of the data. A *p*-value >0.05 indicated the hypotheses of data normality and homogeneity of variance as consistent, and that further parameter testing can be conducted. *P<*0.05 was considered a statistically significant difference [21].

## 3 RESULTS

### 3.1 Phenotypic Characterization of NK Cells

**Figure 1:**
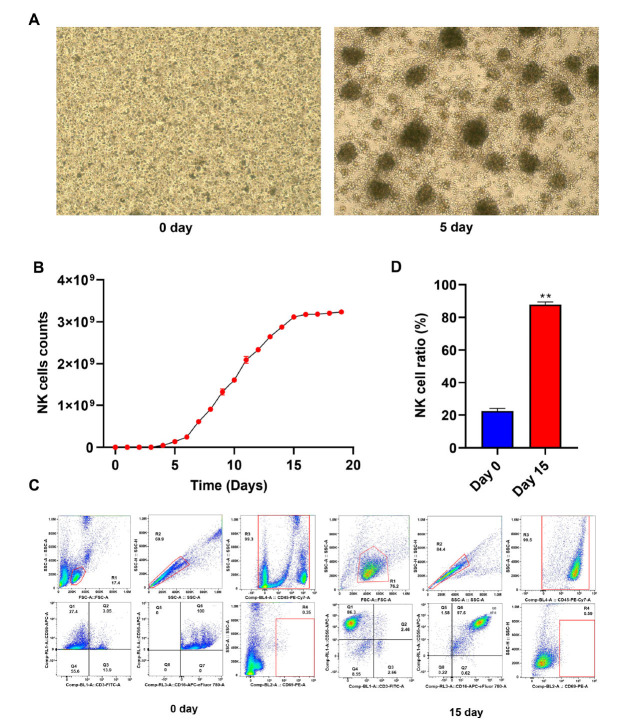
Phenotypic characterization of NK cells. (**A**) The representative optical microscopy images of PBMCs on day 0 and day 5. Scale bar, 200 μm. (**B**) Expansion of NK cells isolated from PBMCs. (**C**) Flow cytometric analysis of NK cells after PBMCs isolation and expansion in the presence of IL-2 for 2 weeks. (**D**) Analysis of the amplification factor of NK cells in (**C**). **Note:** Data represent mean ± SD; **p < 0.01.

NK cells were amplified from PBMCs and purified using magnetic beads for CD56 positive selection. On the 5^th^ day of cultivation, NK cells began to aggregate and grow, and morphological changes were observed (Figure **1A**). Subsequently, NK cells were further cultured under IL-2 stimulation for 2 weeks, and the number of NK cells significantly increased (Figure **1B**). The phenotypic characteristics of NK cells were evaluated using flow cytometry after initial culture and 14 days. The results showed no significant difference (*P*/0.05) between PBMCs and CD16 cultures, while on day 15, the proliferation of the CD3-/56+cell population in NK cell cultures containing IL-2 significantly increased (Figure **1C**), and the amplification factor of NK cells was greater than 300 (Figure **1D**). These results indicated that we successfully isolated and cultured NK cells and activated them with IL-2. After 14 days, their main cell subtype was CD3-/56+.

### 3.2 NK Cells Demonstrated Strong Anti-tumor Activity *In vitro*

**Figure 2:**
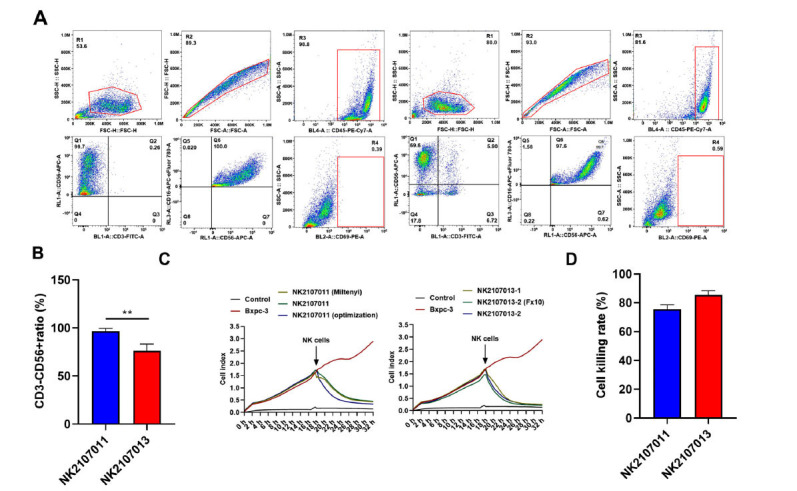
NK cells exhibited potent anti-tumor activity *in vitro.* (**A**) Flow cytometry analysis of NK cells isolated from PBMCs using immunomagnetic beads sorting and expansion in the presence of IL-2 for 2 weeks. (**B**) The killing rate of BXPC-3 by NK cells sorted by immunomagnetic beads. (**C**) Flow cytometry analysis of NK cells isolated from PBMCs without the use of immunomagnetic beads sorting and expansion in the presence of IL-2 for 2 weeks. (**D**) The killing rate of BXPC-3 by NK cells directly isolated from PBMCs. **Note:** Data represent mean ± SD; **p < 0.01.

Before evaluating the lytic activity of NK cells on cancer cell lines, we first assessed the killing effect of NK cells with different CD3-/CD56+ ratios on cancer cells when the ratio of effector to target was 10:1. The results indicated that as long as the proportion of CD3-/CD56+ NK cells was high enough, their killing effect was very significant (Figure **2**). These findings indicated that when the proportion of CD3-/CD56+cell subtypes was high, the cytotoxicity of NK cells was significantly increased.

### 3.3 Fabrication of iRGD-modified NK Cells

**Figure 3:**
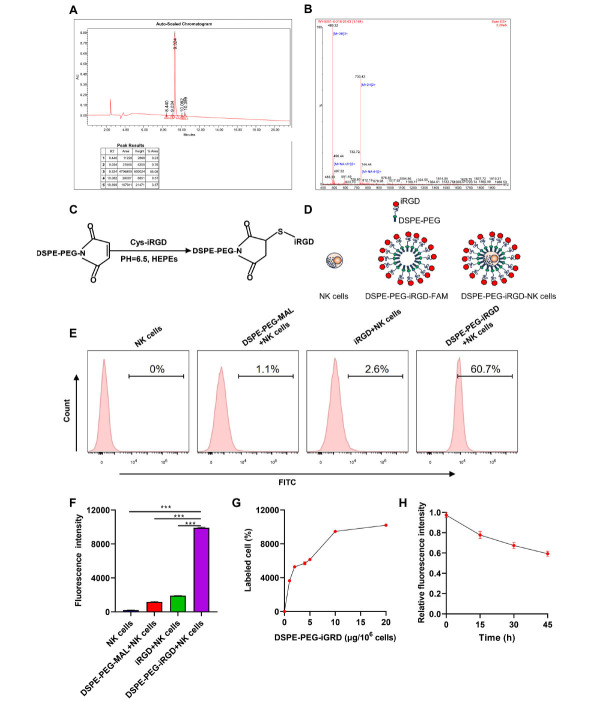
Fabrication of iRGD-modified NK cells. (**A, B**) HPLC and mass spectrometry detected the purity of iRGD. (**C**) Schematic diagram of the synthesis of lipid-conjugated DSPE-PEG-iRGD. (**D**) Schematic of the NK cells’ modification process. (**E**) Flow cytometry histograms of NK cells, DSPE-PEG-MAL+NK cells, iRGD+NK cells, and DSPE-PEG-iRGE-modified NK cells. (**F**) Mean fluorescence intensity of NK cells, DSPE-PEG-MAL+NK cells, iRGD+NK cells, and DSPE-PEG-iRGE-modified NK cells in (**D**). (**G**) Analysis of the percentage of DSPE-PEG-iRGD-FAM-modified NK cells using flow cytometry. (**H**) Analysis of the changes in relative averaged fluorescence intensities of cells modified with DSPE-PEG-iRGD over the culture periods using flow cytometry. **Note:** Data represent mean ± SD; ***p < 0.001.

We used HPLC and mass spectrometry to detect the purity of iRGD, and the results are shown in Figures (**3A** and **B**). To anchor iRGD onto the NK cell membrane, we added cysteine residues at the C-terminus of the peptide. Free sulfhydryl groups allowed iRGD to couple with the maleimide group of DSPE-PEG-Mal *via* Michael addition reaction (Figure **3C**). DSPE-PEG-iRGD-FAM was prepared using the same method for specific experiments. The synthesis mechanism is visually explained using mechanism diagrams in Figure (**3D**). We then evaluated the binding of iRGD to target cells (Figure **3E** and **F**). Flow cytometry showed that using DSPE-PEG-FAM or iRGD alone resulted in almost no encapsulation on the surface of NK cells (iRGD-encapsulated NK cells were figured), and that the obtained DSPE-PEG-iRGD-FAM spontaneously transferred from the solution to the surface of NK cells after overnight co-culture. In addition, we investigated the dose response of DSPE-PEG-iRGD for NK cell binding. DSPE-PEG-iRGD (20 µg) achieved 100% encapsulation of 1×10^6^ activated NK cells (Figure **3G**), demonstrating the effective ability of the cell surface modification platform we used. Since binding stability is a key parameter for cell surface modification, we investigated the cell surface dynamics of DSPE-PEG-iRGD-FAM. After 45 hours of cultivation, the relative fluorescence intensity of DSPE-PEG-iRGD-FAM-modified NK cells decreased to 55%, which is approximately the doubling time of NK cells (Figure **3H**). This result indicated the cell surface modification platform we applied to have good stability.

### 3.4 NK Cells Modified with iRGD Exhibited Enhanced Penetration Capabilities within MCTSs

**Figure 4:**
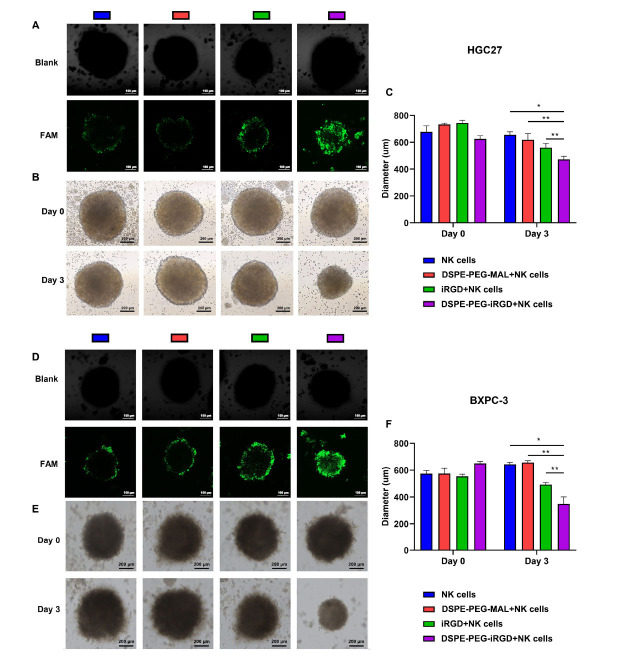
NK cells modified with iRGD possessed superior penetration capacity in MCTSs. (**A**) Confocal microscopy images of HGC27 spheroids treated with CFSE-labeled NK cells, DSPE-PEG-MAL+NK cells, iRGD+NK cells, and DSPE-PEG-iRGE-modified NK cells. Magnification, ×200; scale bar, 100 μm. (**B**) NK cell cytotoxicity against HGC27 tumor spheroids. Representative images of MCTS treated with NK cells, DSPE-PEG-MAL+NK cells, iRGD+NK cells, and DSPE-PEG-iRGE-modified NK cells. Scale bar, 200 μm. (**C**) Growth inhibition assay in HGC27 MCSs. (**D**) Confocal microscopy images of BXPC-3 spheroids treated with CFSE-labeled NK cells, DSPE-PEG-MAL+NK cells, iRGD+NK cells, and DSPE-PEG-iRGE-modified NK cells. Magnification, ×200; scale bar, 100 μm. (**E**) NK cell cytotoxicity against BXPC-3 tumor spheroids. Representative images of MCTSs treated with NK cells, DSPE-PEG-MAL+NK cells, iRGD+NK cells, and DSPE-PEG-iRGE-modified NK cells. Scale bar, 200 μm. (**F**) Growth inhibition assay in BXPC-3 MCSs. **Note:** Data are represented as mean ± SD; *p < 0.05, **p < 0.01.

We created MCTSs using HGC27 cells and BXPC-3 cells. Research has shown NK cells delivered alone or in combination with DSPE-PEG-MAL to have the lowest permeability to MCTSs, with only weak signals detected at the periphery of the MCTSs. It is worth noting that iRGD modification enabled NK cells to penetrate the core of MCTSs within 20 hours (Figure **4A** and **D**). To evaluate the cytotoxicity of infiltrating NK cells, we treated HGC27 and BXPC-3 MCTS with different groups for 48 hours, and then continued to incubate them in fresh medium for 24 hours. Representative images showed that the use of NK cells alone or NK cells co-delivered with DSPE-PEG-MAL could not effectively inhibit MCTS proliferation, although slight inhibition was observed. However, we observed that in the iRGD-modified group, MCTS proliferation was significantly inhibited, the spherical structure was almost destroyed, and some dissociated cells were distributed around the edges of the MCTSs (Figures **4B**, **4C**, **4E** and **4F**). These *in vivo* studies have preliminarily confirmed our hypothesis that iRGD enhances the ability of NK cells to infiltrate tumors more effectively.

### 3.5 NK Cells Modified with iRGD Potently Inhibited Tumor Growth in Solid Tumor Xenograft Mouse Model

**Figure 5:**
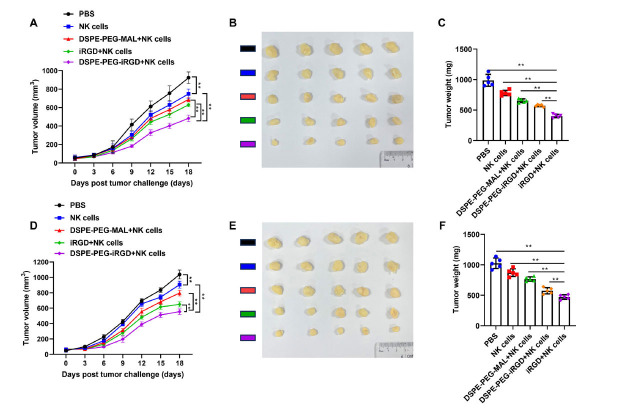
iRGD-modified NK cells effectively controlled tumor growth. (**A, D**) The tumor volume of the mice treated with NK cells modified with or without iRGD. (**B, E**) Photos of tumors harvested from mice in all groups on day 18 after tumor inoculation. (**C, F**) The tumor tissue weight in different groups at the endpoint of the animal experiment. **Note:** Data are represented as mean ± SD; **p < 0.01.

In order to evaluate whether iRGD-modified NK cells can control tumor growth in the preclinical *in vivo* model of solid tumors, adoptive metastasis was carried out using the xenogeneic mouse model of gastric cancer and pancreatic cancer. Research has shown the NK cell-based therapy to successfully inhibit tumor growth, with iRGD-modified NK cells exhibiting the most significant differences compared to the NK cell group (Figures **5A** and **D**). The weight and volume reduction of continuously growing tumor masses obtained from the NK cell group modified with iRGD further confirmed this (Figures **5B**, **C**, **E** and **F**).

## 4 DISCUSSION

Cancer has always been a long-standing disease in human history and has become the leading cause of death worldwide [22]. NK cells exhibit cytotoxic activity against various types of tumor cells, and certain clinical strategies initially aimed at enhancing T cell toxicity may also activate NK cells [23]. Research reports that the number and function of NK cells in patients with cancers are significantly reduced, and the reduction of tumor-infiltrating NK cells is related to the poor survival rate of advanced cancer, such as hepatocellular carcinoma [5] and triple-negative breast cancer [24]. This highlights the importance of intratumoral NK cells in the immune response against solid tumors. Therefore, due to the anti-tumor ability of NK cells, they are expected to become targets for cancer immunotherapy strategies [25].

However, clinical studies on NK cell-based immunotherapy for solid tumors are rare. Due to the tumor microenvironment (TME) and suppressed immunity in solid tumors, NK cell therapy for solid tumors faces many difficulties, resulting in weak NK cell function, poor tumor transport, and NK cell infiltration into tumors [25]. NK cell infiltration has been observed in primary tumors of solid cancer, as well as in metastatic tumors and tumor-infiltrating lymph nodes. However, although NK cells play a crucial role in tumor immunotherapy, their clinical effectiveness is often limited by their limited ability to infiltrate solid tumors [26]. Most research reports indicate that NK cell infiltration is reduced in malignant tissues compared to corresponding non-malignant tissues [27, 28]. Therefore, enhancing the infiltration of NK cells into solid tumors can significantly inhibit tumor progression.

Our data have revealed the unique phenotype of CD3-/CD56+NK cells. In addition, when the proportion of CD3-/CD56+cell subtypes to target cells HGC27 and BXPC-3 was high, the cytotoxicity of NK cells was significantly increased. The fact that spheres from HGC27 and BXPC-3 cell lines were effectively infiltrated and killed by iRGD-modified NK cells further confirms this. Finally, NK cells modified with internal iRGD demonstrated higher cytotoxic potential in xenograft models of solid tumors.

NK cells can be classified into subgroups CD56^bright^ and CD56^dim^, with CD56 being a key biomarker for NK cell function and classification [29]. The role of CD3^−^CD16^bright^CD56^dim^ and CD3^−^CD16^dim^CD56^bright^ NK cells in the tumor microenvironment of various cancers (including breast cancer, melanoma, prostate cancer, and lung cancer) has been explored [9, 30-32]. The adoptive transfer therapy based on NK cells requires *ex vivo* expansion of NK cells, prolonged *in vivo* survival time, optimal *in vivo* activity, and targeted cytotoxicity against cancer cells. In this study, we cultured and activated NK cells isolated from PBMCs, and successfully identified the immune phenotype of NK cells to be mainly CD3^-^CD56^+^. Functional analysis of NK cells showed the expression of cytotoxicity to be much higher when exposed to solid tumor cells. Our results indicated that these NK cells are resting and have the ability to be easily enhanced. Further research has shown NK cells to significantly kill cancer cells and exhibit strong cytotoxicity.

TPP is a special type of membrane-active peptide (MAP) that efficiently delivers nucleic acids, proteins, or other molecules into multiple cell types through receptor-independent mechanisms, while maintaining cell integrity [33]. According to reports, iRGD is a safe TPP with extravasation and tumor tissue infiltration capabilities [34]. For example, Liu *et al.* showed that a combination of iRGD-antiCD3 and PD-1 blockade could enhance the anti-tumor effect of T cells, indicating this method to have great potential in the treatment of solid tumors [14]. Shao *et al.* demonstrated that integrating iRGD with CIML NK cells could enhance their ability to infiltrate and selectively disrupt MCSs [5]. This is similar to our work, where we used iRGD to enhance the permeability of NK cells, but the difference is that Shao’s work only focused on the role of iRGD-modified CIML NK as a tumor in hepatocellular carcinoma. In our study, we explored new application areas of iRGD in the treatment of diversified solid tumors. We found that iRGD modification allowed NK cells to penetrate deep into the core of MCTSs, and the proliferation of MCTSs in the iRGD-modified group was significantly inhibited. In addition, iRGD-modified NK cells significantly reduced tumor growth in a solid tumor xenograft mouse model.

## 5 LIMITATIONS

However, our research study has involved some limitations. We did not conduct an in-depth analysis of the specificity of iRGD in tumor cells, but randomly selected two cancer cell lines. Although the results showed iRGD-modified NK cells to have significant killing and infiltrating effects on both types of cancer cells, further exploration is needed to determine whether this modification has speci-ficity for more cell lines, which will be our next direction.

## CONCLUSION

In summary, we investigated the application of iRGD in NK cell immunotherapy. Our results indicated that NK cells modified with iRGD exhibited enhanced permeability and increased cytotoxicity in MCTS. This combination method may significantly improve the effectiveness of NK cell immunotherapy for different types of solid tumors.

## Data Availability

The datasets used and/or analyzed during the current study will be available from the corresponding author upon reasonable request.

## LIST OF ABBREVIATIONS

NK: Natural Killer
ILCs: Innate Lymphocytes
MHCs: Major Histocompatibility Complexes
TPP: Tumor Penetrating Peptide
HPLC: High-pressure Liquid Chromatography
DSPE-PEG-iRGD: 1, 2-Distearoyl-snglycero-3-phosphoethanolamine-Poly(ethyleneglycol)-iRGD
MCTSs: Multicellular Tumor Spheroids
IL-2: Interleukin-2
WHO: World Health Organization
ACT: Adoptive Cell Therapy
T cells: T Lymphocytes
IL-21: Interleukin-21
PBMCs: Peripheral Blood Mononuclear Cells
C-iRGD: Ac-CCRGDKGPDC-NH2
C-iRGE: Ac-CCRGEKGPDC-NH2
DSPE-PEG-Mal: DSPE-PEG-Maleimide
MALDI-TOF MS: Matrix-assisted Laser Desorption/Ionization-time-of-flight Mass Spectrometry
CFSE: Carboxyfluorescein Succinimidyl Ester
SD: Standard Deviation
ANOVA: One-way Analysis of Variance
TME: Tumor Microenvironment
MAPs: Membrane-active Peptides
